# Adenosine and Its Receptors in the Pathogenesis and Treatment of Inflammatory Skin Diseases

**DOI:** 10.3390/ijms25115810

**Published:** 2024-05-27

**Authors:** Luxia Chen, Xuan Lei, Karsten Mahnke

**Affiliations:** Department of Dermatology, University Hospital Heidelberg, Im Neuenheimer Feld 440, 69120 Heidelberg, Germany; luxia.chen@med.uni-heidelberg.de (L.C.);

**Keywords:** adenosine receptors, inflammation, psoriasis, hyperplasia, immunosuppression

## Abstract

Inflammatory skin diseases highlight inflammation as a central driver of skin pathologies, involving a multiplicity of mediators and cell types, including immune and non-immune cells. Adenosine, a ubiquitous endogenous immune modulator, generated from adenosine triphosphate (ATP), acts via four G protein-coupled receptors (A_1_, A_2A_, A_2B_, and A_3_). Given the widespread expression of those receptors and their regulatory effects on multiple immune signaling pathways, targeting adenosine receptors emerges as a compelling strategy for anti-inflammatory intervention. Animal models of psoriasis, contact hypersensitivity (CHS), and other dermatitis have elucidated the involvement of adenosine receptors in the pathogenesis of these conditions. Targeting adenosine receptors is effective in attenuating inflammation and remodeling the epidermal structure, potentially showing synergistic effects with fewer adverse effects when combined with conventional therapies. What is noteworthy are the promising outcomes observed with A_2A_ agonists in animal models and ongoing clinical trials investigating A_3_ agonists, underscoring a potential therapeutic approach for the management of inflammatory skin disorders.

## 1. Introduction

Many intercellular signals maintain homeostasis in tissues and organs, with adenosine being one of the earliest identified regulators of these signals, governing various physiological and pathological processes. Adenosine was first discovered as a potent vasodilator in 1929 by Drury and Szent-Gyorgi [1]. Described as a “retaliatory metabolite” [2], adenosine is released in response to conditions such as hypoxia, metabolic stress, or injury, promoting the clearance of these noxious stimuli. In particular, metabolic stress, i.e., the shortage of nutrients, reduced availability of oxygen, and/or production of radical oxygen species in a cellular environment during inflammation and tumor growth, has caught attention lately. In this situation, immune cells have to compete for resources to generate energy with each other or with tumor cells. Here, adenosine, other than classical chemo- and cytokines, may connect the metabolic status of the cells with the immune response [3], as it is a derivative of the major energy carrier ATP. Adenosine is a degradation product of ATP as well as a precursor for ATP, which is generated by phosphorylation, therefore, its production and degradation is directly linked to the energy turnover in cells. Based on these mechanisms, adenosine receptors are able to sense the metabolic status of cells and can activate intracellular pathways to keep the energy supply in cells in balance. For instance, glucose uptake [4,5] and the induction of antioxidative enzymes, as well as the blockade of ROS production [6], all of which promote cell survival [7], are regulated by adenosine.

In skin, the increased accumulation of adenosine is observed in various inflammatory skin disorders. Notably, adenosine serves as an endogenous regulator of inflammatory processes, facilitating the transition from inflammation to healing [8]. Pathological changes in, or pharmacological manipulation of, adenosine metabolism or adenosine receptor expression and/or function(s), might play a role in both, the pathogenesis, and the therapy of inflammatory skin diseases.

Tempering receptor signaling and pro-proliferative pathways by small-molecule inhibitors have emerged from cancer therapies. Here, more than 80 different chemicals or proteins mimicking the binding of ligands to specific enzymes, blocking the respective enzyme, have been identified [9]. Similar to these small-molecule inhibitors in cancer, molecules that are able to bind adenosine receptors without triggering their signaling, are known, and they may be useful to block adenosine-related intracellular pathways [10]. Furthermore, molecules involved in the production of adenosine, such as the ectonucleotidase CD73, may be targets for intervention. As already defined in tumors, CD73 acts as the “checkpoint” [11], and antibodies against CD73 may be able to cease its adenosine generating capacity, even in skin.

This review explores the metabolic changes induced by aberrant adenosine triphosphate (ATP) and adenosine levels in inflamed skin, the receptors that mediate the pathological and pharmacological effects of adenosine and their role in inflammatory skin diseases, as well as the therapeutic potential of targeting adenosine and its receptors. Since small-molecule inhibitors have emerged as a cutting-edge therapeutic approach for many kinds of tumors and skin diseases, adenosine and its receptors have also become targets of mechanistic research and therapeutic intervention.

## 2. Background of Adenosine and Its Receptor

### 2.1. Biochemistry of Adenosine: Source, Regulation, and Uptake

Adenosine is produced in the extracellular space through the sequential dephosphorylation of adenine nucleotides (ATP and adenosine diphosphate (ADP)) to adenosine. Intracellular ATP is exported out of cells via several transporters, including connexin-43, progressive ankylosis protein homolog (ANK) 6, pannexin-1, and pannexin-3 [8]. The extracellular adenosine precursors (i.e., nucleotide ligands), including ATP, ADP, and uridine triphosphate (UTP), are recognized by P2 receptors to induce specific signaling pathways [12].

Based on their molecular structure, P2 receptors are divided into two families: P2X and P2Y receptors. P2X receptors, which are ligand-gated ion channels, are named sequentially from P2X_1_ to P2X_7_. Upon exposure to extracellular ATP, they elicit a flow of cations (Na^+^, K^+^, and Ca^2+^) through the plasma membrane [13]. Differently, the P2Y receptors belong to a subclass of the superfamily of G-protein-coupled receptors (GPCRs), featuring seven transmembrane domains, with eight subtypes described so far [14].

**Figure 1:**
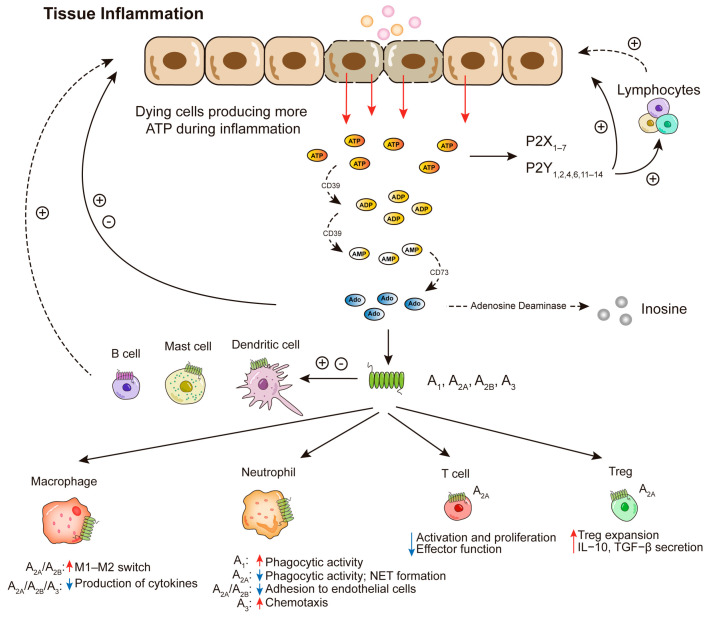
Adenosine production and its impact on regulating the function of inflammatory cells via adenosine receptors. In inflamed tissue, more ATP is generated or released by dying cells. Extracellular ATP binds to P2 receptors (P2X and P2Y) to exert its effects. Degraded sequentially by the nucleotidases CD39 and CD73, ATP converts to ADP and AMP, ultimately leading to the production of adenosine. Adenosine engages with four adenosine receptors (A_1_, A_2A_, A_2B_, and A_3_), modulating immune response. Adenosine deaminase is subsequently responsible for deamination of extracellular adenosine to inosine. Both P2 receptors and adenosine receptors contribute to the complex modulation to leukocyte and tissue cell functions. Upon adenosine binding to the different receptor subtypes, the functions of leukocytes are enhanced or reduced, represented by red and blue arrows, respectively. The impacts of adenosine and its receptors on macrophages, neutrophils, T cells, and Tregs are depicted. Additionally, extracellular adenosine exerts its effect by interacting with adenosine receptors on other immune cells, such as dendritic cells, B cells, and mast cells. Ado, adenosine; ADP, adenosine diphosphate; AMP, adenosine monophosphate; ATP, adenosine triphosphate; Treg: regulatory T cell.

Extracellular ATP is rapidly degraded, with a very short half-life of approximately one to five min, depending on the tissue [15,16]. ATP can be sequentially dephosphorylated to adenosine by cell surface-expressed enzymes (ectoenzymes) “ecto-nucleoside triphosphate phosphohydrolase” (CD39) and “ecto-5′nucleotidase” (CD73), or by soluble enzymes in blood or other extracellular fluids (Figure 1). Once generated and having engaged its receptors (adenosine receptors or P1 receptors), adenosine can either be internalized via nucleoside transporters or deaminated to inosine by adenosine deaminase (ADA). In humans, inosine is further deaminated to uric acid or taken up directly by cells through specific nucleoside transporters (ENT1 and ENT2), whereby its re-phosphorylation may be used to replenish the adenosine content in cells [8,17].

Under normal physiological conditions, ATP and adenosine concentrations remain low, regulated by the activity of enzymes and transporters. However, in various pathological states, such as inflammation, injury, and hypoxia, these molecules can be released into the extracellular environment. Here, they engage different receptors, initiating a diverse range of signaling pathways. This activation mediates various physiological processes, including proliferation, differentiation, migration, and cell death, making it difficult to clearly single out defined adenosine-mediated effects in a huge organ, such as the skin.

### 2.2. Overview of Adenosine Receptors (A_1_, A_2A_, A_2B_, and A_3_) and Their General Functions

The adenosine receptors (or P1 receptors) comprise four G protein-coupled receptor subtypes, A_1_, A_2A_, A_2B_, and A_3_, which are named based on the order of their discovery. These receptors are able to modulate adenylyl cyclase (AC) activity: A_2A_ and A_2B_ function as stimulators of AC via their Gs subunit, whereas A_1_ and A_3_ act as inhibitors via their Gi subunits. Consequently, A_2A_ and A_2B_ receptors elevate cyclic adenosine monophosphate (cAMP), whereas A_1_ and A_3_ receptors reduce cAMP levels. cAMP subsequently activates protein kinase A (PKA), which can phosphorylate and activate cAMP-responsive element-binding protein (CREB). Phosphorylated CREB can mediate gene expression directly by interacting with the gene promoter or indirectly by competing with nuclear factor-κB (NF-κB) or other transcription factors, thus inhibiting the expression of genes encoding proinflammatory cytokines [18,19]. Nonetheless, the reduced levels of cAMP triggered by A_1_ and A_3_ receptor activation result in PKA inhibition. Additionally, A_1_ and A_3_ receptors are coupled to phospholipase C (PLC) via Gq proteins, leading to increased calcium levels and the activation of protein kinase C (PKC) [20]. Accumulating evidence supports the fact that A_3_ receptor activation mediates anti-inflammatory activity by regulating phosphoinositide-3-kinase–protein kinase B/Akt (PI3K/Akt) and NF-κB signaling pathways [20,21].

## 3. Molecular Mechanisms of Adenosine

### 3.1. Adenosine-Induced Actions in Skin Cells

#### 3.1.1. Adenosine in Keratinocytes and Fibroblasts

Keratinocytes, key pathogenic cells in both the initiation and maintenance phases of inflammatory skin diseases, respond to multiple triggers as part of the innate immune system. Stressed keratinocytes promote the activation of dendritic cells by releasing peptides and produce copious chemokines to recruit leukocytes as well as other inflammatory mediators to amplify inflammation [22]. Furthermore, the crosstalk between keratinocytes and immune cells fosters abnormal phenotypes of keratinocytes that are characterized by disrupted intercellular binding or by the hyperplasia of immature keratinocytes, as observed in psoriasis.

The expression of adenosine receptors has been demonstrated in keratinocytes, with the A_2B_ receptor being the major receptor. However, other adenosine receptors may be expressed too; however, conflicting findings have been reported regarding their expression. Probably due to their low levels, a definitive expression pattern could not be established [23,24]. As for the A_2B_ receptors, studies have indicated that human keratinocytes express predominantly A_2B_ receptors without detectable levels of A_1_, A_2A_, or A_3_ receptor mRNA [12]. Additionally, murine keratinocytes, either derived from primary cultures or as MSC-P5 cell lines, exhibit the strongest expression of A_2B_, albeit accompanied by lower expressions of A_2A_ and A_3_ receptors [25].

The findings of the effects of adenosine on the proliferation of epidermal cells are contradictory as well, probably depending on the differential expression of different combinations of adenosine receptors. Previous studies have highlighted the stimulatory effects of adenosine in human and murine keratinocytes and in melanocytes, mediated by A_2A_ and A_2B_ receptors [25,26], while A_3_ stimulation arrests proliferation [26]. These opposing effects of adenosine are further substantiated by a recent study on human keratinocytes (normal human epidermal keratinocytes, NHEK), reporting the presence of both A_2A_ and A_2B_ receptors in those cells. The study identifies that adenosine inhibits keratinocyte proliferation via A_2B_ receptors while stimulating their proliferation via A_2A_ receptors. The anti-proliferative effect of A_2B_ was mediated via modulation of intracellular calcium increase and p38 phosphorylation, without the involvement of Gs or cAMP [23]. Thus, adenosine may have pro- and anti-inflammatory effects at the same time, depending on the receptor pattern expressed by keratinocytes, and in inflammatory conditions, such as psoriasis, the expression of adenosine receptor subtypes is also altered, contributing to the pathology of epidermal hyperplasia.

Adenosine plays a role in enhancing wound healing, potentially influencing fibroblasts. Similar to its various effects on keratinocyte proliferation, adenosine and its receptors have contradictory impacts on collagen and matrix protein production by fibroblasts. Acting through the A_2A_ receptors, adenosine directly stimulates collagen production by dermal fibroblasts and stimulates the production of factors like interleukin (IL)−13 and connective tissue growth factor, which amplifies collagen synthesis. In contrast, A_2B_ receptor activation results in the inhibition of collagen production, at least in some tissue-specific fibroblasts (such as cardiac fibroblasts) [27]. However, human fibroblasts from systemic sclerosis (SSc) patients and fibroblasts in a mouse fibrosis model revealed elevated A_2B_ expression compared to healthy controls, and A_2B_ receptor activation functions as a potential pro-fibrotic regulator in dermal fibrosis [28].

#### 3.1.2. Adenosine in Melanocytes

Melanocytes originating from the neural crest are melanin-producing cells in the skin, hair, and eyes [29]. Knowledge about adenosine receptors on melanocytes is limited [12]. However, an earlier study has shown that adenosine A_2B_ receptors are upregulated by phenolic skin-bleaching agents, the activation of which led to melanocyte apoptosis [30].

Compared to the direct impact of adenosine on melanocytes, cAMP signaling is more intricately associated with differentiation and the pigmentation of melanocytes. The activation of the melanocortin-1 receptor (MC1R), induced by the α-melanocyte stimulating hormone (α-MSH), leads to the activation of the cAMP signaling pathway. Increased cAMP activates PKA, which phosphorylates CREB, a crucial factor for melanocyte differentiation and pigment production [31]. Consequently, many drugs or genetic alterations that modify the cAMP signaling pathway are associated with pigmentation abnormalities and (or) melanoma development. For instance, impaired cAMP signaling caused by single nucleotide polymorphisms in MC1R results in impaired melanogenesis, leading to red hair color and a fair skin phenotype in humans [32]. Without the effective protection of melanin, those individuals carry a high risk of skin cancer due to their increased susceptibility to the cytotoxic effects of UV radiation [33]. Similarly, mice with non-functional MC1R and deficient cAMP signaling are more susceptible to developing melanoma [34]. Interestingly, sex steroids such as estrogen have been shown to induce cAMP signaling in melanocytes, increasing melanin synthesis [35].

Considering the bi-phasic modulation of adenosine on cAMP signaling, adenosine and its receptors are expected to play a role in regulating the cytophysiology of melanocytes and melanogenesis. However, research on the effect of adenosine on benign melanocytes is lacking. More studies have focused on melanoma cells, identifying the expression of A_1_, A_2A_, A_2B_, and A_3_ adenosine receptors in the human malignant melanoma A375 cell line [36]. In this melanoma cell line, adenosine has been found to reduce cell proliferation via A_3_ receptors and promote cell death, probably via A_2A_ receptors [26]. The inhibition of the A_3_ receptor on cell proliferation was found to be mediated by PI3K phosphorylation [37].

Furthermore, the effect of adenosine on melanogenesis was investigated in several studies, using B16 melanoma cells. It was found that a low concentration of adenosine promotes melanogenesis by increasing melanin and tyrosinase activity and expression, which is the key enzyme for the initial step to generate 4-dihydroxyphenylalanine (DOPA) and melanin, whereas this effect was inhibited by a high dose of adenosine [38]. The bi-phasic activity of adenosine on melanogenesis may be due to the different impacts of specific adenosine receptors on cAMP signaling. Additionally, another study highlighted the enhancement of melanogenesis via activation of A_3_ adenosine receptors. Piclidenoson (IB-MECA or CF101), an agonist of A_3_ adenosine receptors, increased melanin levels in B16 melanoma cells despite decreasing cAMP. The activation of the PI3K/AKT signaling pathway is involved, as inhibition of the pathway abolishes the stimulatory effect of piclidenoson on the melanin level of B16 cells. Similarly, piclidenoson exposure to human skin explants also increased DOPA positive cells and melanin deposition in keratinocytes [39]. Thus, adenosine regulates melanogenesis and the development of melanocyte and melanoma cells through interactions with its receptors and various downstream pathways, emphasizing the necessity for further studies to unravel this complicated network and explore its potential in treating pigmentation disorders and melanoma.

#### 3.1.3. Adenosine in Innate Immune Cells

Adenosine serves as a potent inhibitor of inflammation by modulating the functions of various immune cells, including neutrophils, macrophage/monocytes, dendritic cells, and lymphocytes, particularly in diseases characterized by overactivated immune responses (Figure 1).

Neutrophils are engaged in acute inflammation. Induced by a pleiotropy of different chemotactic factors, such as IL-8, neutrophil-activating peptide (NAP)-2, NAP-3, and lipopolysaccharide (LPS) [40], they infiltrate the skin. The transition of neutrophils from the vasculature to the extravascular space (i.e., skin) is facilitated by adhesive interactions with endothelial cells and the extracellular matrix. The impact of adenosine on neutrophil adhesion to endothelial cells varies across numerous studies. Specifically, it was reported that engagement of A_2A_ and A_2B_ receptors is associated with the reduced adhesion of neutrophils to endothelial cells by inhibiting selectin- and integrin-mediated interactions [41]. Conversely, activation of the A_1_ receptor has been shown to enhance neutrophil adhesion to endothelial cells through α4 integrins [42] because A_1_ receptor-specific pharmacological inhibitors led to reduced chemotaxis of neutrophils [43,44]. Furthermore, studies using knockout mice [45,46] demonstrated that the A_3_ receptor actively facilitates increased chemotaxis in neutrophils. Thus, the involvement of adenosine in neutrophil migration in the skin seems obvious; however, the outcome is critically dependent on the pattern of adenosine receptor expression by the cells.

After transitioning into the locally inflamed tissue, neutrophils act as frontline defenders of innate immunity, engaging in phagocytosis and releasing pro-inflammatory mediators. While the robust response of neutrophils facilitates the destruction of pathogens, it also contributes to skin pathology by the uncontrolled release of reactive oxygen species (ROS) and cytokines, such as IL-17A [47,48]. Previous studies have highlighted the role of neutrophils in the onset of several autoimmune and inflammatory diseases, such as psoriasis, systemic lupus erythematosus (SLE), rheumatoid arthritis (RA), inflammatory bowel diseases, atherosclerosis, and others [49]. Specifically, in psoriatic lesions, neutrophils infiltrate the dermis and epidermis, leading to the formation of Kogoj or Munro’s microabscesses, which serve as key pathologic diagnostic clues for psoriasis [50]. Adenosine, in this context, exerts a dual role on the neutrophil functions, mediated by A_1_ and A_2A_ adenosine receptors. Specifically, the A_1_ adenosine receptor enhances the neutrophil phagocytic activity and induces the generation of ROS, whereas activation of the A_2A_ adenosine receptors produces opposite effects [51]. Interestingly, adenosine also impacts Neutrophil Extracellular Traps (NETs), which are chromatin filaments coated with pro-inflammatory and effector molecules released by neutrophils. NET formation restricts pathogen spreading and is linked to autoimmune diseases due to its degradation of the extracellular matrix and amplification of the immune response with its pro-inflammatory molecules. Adenosine, via activation of the A_2A_ adenosine receptors, attenuates NET formation and suppresses the ‘NETosis’ activity of NETs [52,53].

Macrophages are essential in two main aspects: destroying microbes and initiating immune responses. The latter is mostly mediated by presenting antigens to T cells and orienting adaptive immune response through cytokine production and other mediators. Macrophages are broadly classified into M1 (classical) and M2 (alternatively activated) subtypes. M1 macrophages are essential in releasing pro-inflammatory cytokines, oxidants, nitric oxide, and other small-molecule mediators of tissue injury. In contrast, M2 macrophages contribute to terminating inflammation and promoting wound healing [54]. Adenosine, via A_2A_, A_2B_, and A_3_ adenosine receptor activation, suppresses the production of pro-inflammatory cytokines in M1 macrophages [51]. In parallel, adenosine enhances the expression of anti-inflammatory mediators such as IL-10 and vascular endothelial growth factor (VEGF) via A_2A_ and A_2B_ adenosine receptors. Furthermore, stimulation of both A_2A_ and A_2B_ receptors induces a phenotypic switch of macrophages from an M1 to a modified M2 phenotype. This switch contributes to sustaining an anti-inflammatory environment and promotes wound healing by facilitating the production of angiogenic and profibrotic cytokines [8,55].

#### 3.1.4. Adenosine in T Cells

Adenosine exerts suppressive effects on CD8^+^ T cell activity via signaling through A_2A_ and/or A_2B_ receptors. Specifically, A_2A_ receptor signaling impairs T cell activation, proliferation, and IL-2 release by polarized cytotoxic CD8^+^ T cells [51] by inhibiting “neurogenic locus notch homolog protein 1” (Notch1) activity and the mammalian target of rapamycin complex (mTORC1) pathways [56,57]. In parallel, the A_2A_ adenosine receptor diminishes the production of inflammatory cytokines in CD4^+^ T cells. Additionally, A_2A_ signaling upregulates immune-checkpoint molecules such as T cell immunoglobulin and mucin domain-containing protein 3 (TIM3), cytotoxic T-lymphocyte-associated protein 4 (CTLA-4), and programmed cell death protein 1 (PD-1) on effector T cells [58,59]. Furthermore, A_2A_ adenosine receptor activation inhibits the polarization of naïve T cells into T helper 1 cell (Th1) and Th2 cells and attenuates the production of interferon (IFN)-γ and IL-4 [60]. Adenosine has been reported to facilitate the differentiation of Th17 by engaging A_2B_ receptor on dendritic cells (DCs) and inducing IL-6 production [61,62]. Nonetheless, during the early stage of autoimmune uveitis, non-selective activation of adenosine suppresses both Th1 and Th17 responses [63].

Notably, A_2A_ receptor signaling is playing a key role in the function of regulatory CD4^+^ T cells (Tregs), a subtype that is crucial for maintaining tolerance to self-antigens and exerting immunosuppressive effects during inflammation. Adenosine, generated by the ectoenzymes CD39 and CD73, which are strongly expressed by Tregs [64], is a pivotal component in the immunosuppressive repertoire of Tregs, leading to dampening the functionality of effector T cells. A_2A_ adenosine receptors expressed by Tregs promote their expansion, enhance the secretion of immunosuppressive cytokines, such as transforming growth factor (TGF)-β and IL-10, and upregulate co-inhibitory receptors such as PD-1, CTLA-4, and lymphocyte-activation gene 3 (LAG3) [65,66]. This mechanism contributes to a self-amplification loop within the skin, facilitating processes such as accelerating wound healing, immune tolerance to skin commensal microbes, maintenance of skin homeostasis, and orchestrating stem cell-mediated hair follicle regeneration [67,68]. Using the murine model of contact hypersensitivity (CHS), it has been revealed that tissue-homing CD73-expressing Tregs in blood and lymph nodes are crucial for the regulation of skin inflammatory reactions and facilitating tolerance induction [69]. Moreover, the identification of skin-resident memory Tregs has sparked inquiries into their function in maintaining skin homeostasis [70].

### 3.2. Interaction with Other Inflammatory Mediators and Pathways

The expression of adenosine receptors is modulated by a variety of stimuli. Particularly, agents activating NF-κB, such as tumor necrosis factor (TNF)-α, IL-1, and endotoxin, induce the upregulation of A_2A_ receptors, serving as a feedback mechanism to inhibit inflammation. This effect is corroborated by studies in psoriasis patients, where increased A_2A_ receptor expression on peripheral white blood cells (PBMCs) is hindered following anti-TNF-α therapy. Moreover, TNF-α and other pro-inflammatory cytokines enhance adenosine receptors functionality by preventing desensitization. Conversely, IFN-γ decreases both the expression and functionality of A_2A_ receptors [8].

Imiquimod induces psoriasis in mouse models through the activation of toll-like receptor (TLR) 7/8-mediated production of proinflammatory mediators [71]. However, despite the induced IL-1β, IL-6, IL-8, and TNF-α expression in keratinocytes, imiquimod inhibits A_2A_ receptor signaling during this process. This inhibition occurs because imiquimod acts as an antagonist of the A_2A_ receptor, independent of its TLR7/8 activation function. Consequently, the transcriptional activation of proinflammatory cytokines is attributed to the reduced cAMP resulting from A_2A_ antagonism and TLR7/8-mediated activation of the NF-κB signaling cascade [72,73]. Thus, while A_2A_ receptor activation typically serves as an intrinsic regulator of immune reactions, it can also be modulated by external pathogenic agents, leading to an augmentation of inflammatory responses.

Additionally, TNF-α also upregulates A_3_ adenosine receptor expression, as it is elevated in PBMCs from patients suffering from rheumatoid arthritis, psoriasis, and Crohn’s disease, whereas A_3_ adenosine receptors are low or absent in cells from healthy donors [74]. In line with this, anti-TNF-α treatments in RA patients have been observed to reduce A_3_ receptor expression, which correlates with therapeutic response. It further suggests the involvement of NF-κB in this upregulation [75].

## 4. Adenosine in Skin Pathology

Inflammatory skin diseases, such as atopic dermatitis and psoriasis, are characterized by an inflammation-mediated pathology that is predominantly localized in skin lesions. Both, innate and adaptive immune mechanisms, contribute to the complex inflammatory processes observed.

### 4.1. Psoriasis

Psoriasis is a chronic inflammatory skin disease characterized by its relapsing and remitting nature, affecting both skin and joints. The immune mechanism underlying its pathology is driven by the dysregulation of Th1 and Th17 cells, which are activated by myeloid DCs and their release of IL-23 and IL-12. This is paralleled by the accumulation of neutrophils [24].

**Figure 2:**
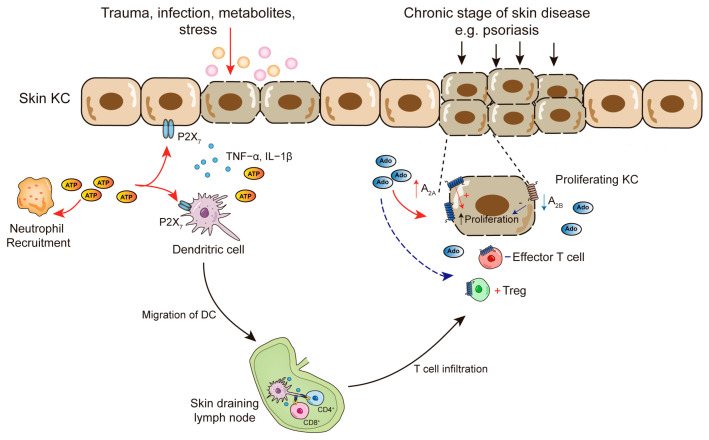
The role of ATP and adenosine in the pathogenesis of different phases of psoriasis. In the initial phase of psoriasis, various factors such as trauma, infection, accumulation of cell metabolites, or stress triggers ATP release. Extracellular ATP stimulates the immune cells and activates the inflammation by binding to the P2X_7_ receptor in keratinocytes and in dermal DCs. This further stimulates the production of pro-inflammatory (TNF-α, IL-1β) cytokines and migration of DCs to the skin-draining lymph nodes, where they present antigens to CD4^+^ and CD8^+^ T cells. In the later phase of psoriasis, adenosine plays a role in modulating keratinocyte proliferation. Endogenous adenosine contributes to hyperkeratosis by promoting the proliferation of keratinocytes via engagement of A_2A_ receptor, which is overexpressed in psoriatic patients. Upon infiltration of T cells into the skin, adenosine suppresses the function of effector T cells while enhancing the activity and expansion of Tregs. ATP: adenosine triphosphate; Ado: adenosine; TNF-α: tumor necrosis factor-α; IL-1β: interleukin-1β; DCs: dendritic cells; KC: keratinocytes; Tregs: regulatory T cells.

Extracellular ATP is one of the alarmins that have been proposed as initiator-events for psoriasis. Following trauma or infection, ATP is released from damaged or necrotic cells, or it may also be released from the sympathetic nervous system under stress (Figure 2). During this stage, ATP initiates signaling directly through its receptors rather than generating extracellular adenosine. Indeed, in the imiquimod-psoriasis mouse model, as previously mentioned, TLR7 activation by imiquimod in the skin appears to inhibit adenosine receptors, suggesting a propensity to trigger a stronger immune response during the initial stage of psoriasis [72].

P2X_7_ receptors are highly upregulated in psoriatic lesions in both humans and in models, serving as an early trigger of psoriasis pathogenesis in a susceptible microenvironment. P2X_7_ receptor signaling is closely associated with psoriasiform dermatitis, particularly through its functions in IL-23-mediated inflammation. This signaling is dependent on the IL-1β/NLRP3 inflammasome and IL-17-expressing neutrophils. Notably, the ATP analog (BzATP), together with an E-NTPDases inhibitor, was able to initiate the development of a full psoriasiform response, which is eliminated by the treatment with anti-Ly6G antibody, highlighting the essential role of P2X_7_-activated neutrophils in the onset of psoriasiform dermatitis [76]. In line with this, P2X_7_ receptors are also reported to activate NF-κB and the IL-23/IL-17 axis and induce DC17 differentiation and Th17 responses [77].

At the chronic stage of psoriasis, the immune circuits that normally participate in the regulation of skin homeostasis become abnormally activated and amplified, leading to an excessive and rapid growth of the epidermal layer of the skin [51]. Elevated adenosine production and its release are one mechanism to exert immunosuppressive effects and limit the extent of inflammation to the local sites of disease (Figure 2). During this process, adenosine also promotes the proliferation of keratinocytes in the lesional skin of psoriasis. Importantly, psoriatic epidermis exhibits a deregulated adenosine receptor expression profile, with reduced A_2B_ but increased A_2A_ expression. In this way, adenosine augments keratinocyte proliferation in psoriatic skin lesions by increasingly engaging the rather stimulatory A_2A_ receptors, as opposed to the presumably inhibitory acting A_2B_ receptors [24].

### 4.2. Skin Inflammation and Anergy

The CHS model is a mouse model of allergic contact dermatitis that is employed in many drug studies. It is an antigen-specific T cell response with classical “sensitization” and “challenge” phases. In the “sensitization” phase, DCs recognize and present antigenic peptides to T cells, and inflammation is induced by challenge, i.e., by a second antigen application [78]. A critical role of ATP in cutaneous inflammation is supported by the fact that CHS responses are inhibited in P2X_7_ ^−/−^ mice and potentiated in the presence of ATPγS, an ATP analog [76,79,80]. This is further evidenced by the severe impairment of P2X_7_-deficient DCs in releasing IL-1β and stimulating antigen-specific T cells [80], pointing to the pivotal role of the P2X_7_ receptor in mediating IL-1β release, which is a critical factor for sensitization.

However, adenosine’s engagement with its receptors on various immune cells facilitates the alleviation of inflammatory responses and maintains tolerance in contact hypersensitivity reactions. For example, CD73-deficient mice, which failed to generate CD73-derived extracellular adenosine, also failed to induce tolerance against 2,4-dinitrofluorobenzene (DNFB) by 2,4-dinitrothiocyanobenzene (DNTB). In particular, this is mediated by the less-activated functional state of CD73^−/−^ Tregs, which express Ki67, CTLA4, C-C Chemokine Receptor (CCR) 4, CD103, CCR6, and CD49b in the skin-draining lymph nodes [69]. In addition, CD73^−/−^ DCs did not promote the differentiation of effector T cells; rather, a hyporeactive phenotype of T cells with an upregulation of anergic markers, such as “N-Myc Downstream Regulated 1” (NDRG1) and “Early Growth Response Protein 2” (EGR2), was induced [81].

Activation of the A_2A_ and A_2B_ pathways contributes to the amelioration of skin inflammation, as topically applied agonists on the ear before sensitization and challenge reduce ear swelling in mice in a CHS model. This was mainly mediated by fewer skin migratory DCs and fewer activated T cells [78]. However, as stated before, four different adenosine receptors have different capacities to convey immunostimulatory and/or immune suppressive signals; therefore, the dynamic changes in adenosine receptor expression during sensitization, challenge, and tolerance induction and their specific contribution to the initiation and termination of inflammation remain to be investigated, to develop “targeted” intervention strategies.

## 5. Clinical Evidence and Studies

Research on adenosine receptor agonists and antagonists involves various studies and clinical trials, focusing on conditions such as psoriasis, eczema, and allergic contact dermatitis. Considering the critical role of ATP and P2X_7_ receptors in skin pathology, studies on the modulation of P2X_7_ receptors, not just adenosine receptors, are also discussed in this review. These investigations have explored the therapeutic potential of modulating the receptors in animal models and clinical trials, aiming to understand their efficacy and mechanisms in treating exemplary skin diseases (Table 1 and Table 2).

### 5.1. P2 Receptors

Since P2X_7_ receptor signaling plays a vital role in initiating inflammation, P2X_7_ receptor modulation has emerged as a potential therapeutic strategy for multiple inflammatory conditions. The first P2X_7_ receptor antagonist (AZD9056) entered the clinic for rheumatoid arthritis and Crohn’s disease, targeting peripheral inflammatory disorders. More recent P2X_7_ antagonists in clinical trials with higher blood–brain barrier permeability and therefore increased ability to enter the central nervous system have spurred investigations focused on neuroinflammatory indications such as depression, Alzheimer’s disease, and Parkinson’s disease [87].

Currently, the potential for P2X_7_ receptor blockade in the treatment of skin diseases is only investigated in pre-clinical mouse models. These effects mainly rely on the ability of the antagonists to inhibit the release of IL-1β by various cells. For instance, the use of the P2X_7_ receptor antagonist A438079 effectively blocks the psoriasiform dermatitis and inflammatory response induced by a combination of ATP analogs and E-NTPDase inhibitors [76]. Similarly, mice treated with A438079 impair croton oil-induced edema, IL-1β production, and neutrophil infiltration [82]. P2X_7_ blockade with its antagonist KN-6, together with the ATP-degrading enzyme apyrase, if applied before sensitization, impairs skin inflammation in a CHS model, presumably through the prevention of IL-1β secretion by DCs [80]. However, unlike neurology, clinical trials with P2X_7_ antagonists in skin diseases have not been initiated yet. Nevertheless, P2X_7_ represents a potential biomarker and target for the treatment of various skin disorders, and further studies are required to assess the clinical value of the P2X_7_ blockade.

### 5.2. A_1_ Adenosine Receptors

As the first member of the adenosine receptor family to be discovered, the A_1_ receptor has been implicated in numerous diseases, yet it remains poorly targeted for clinical purposes. The A_1_ receptor is responsible for various inhibitory effects of adenosine, as it is antiepileptic, acts as a sleep inducer, and couples inositol-1,4,5-trisphosphate (IP3) generation and K^+^ channels in the central nervous system (CNS) and heart.

The natural antagonists of A_1_ receptors include caffeine, which accounts for its excitatory effects by counteracting the inhibiting effects of A_1_ signaling in the CNS. In the repertoire of inflammatory conditions, most studies involving A_1_ receptor manipulation focus on multiple sclerosis (MS), a neuroinflammatory autoimmune disease, with experimental autoimmune encephalomyelitis (EAE) as the corresponding animal model. EAE induction in A_1_ receptor-deficient mice demonstrated more severe symptoms compared to wild type mice, with elevated microglial activation, pro-inflammatory cytokine gene expression patterns, and demyelination, indicating a safeguarding role of A_1_ receptor activation in normal conditions [88]. Moreover, local administration of the A_1_ agonist 2-Chloro-N6-cyclopentyladenosine (CCPA) demonstrates a protective effect against EAE in wild type but not A_1_ receptor-deficient mice [53,89]. Dexamethasone (DEX), a commonly used medication to treat MS, also attenuates inflammation in EAE mice by upregulating A_1_ expression due to decreased levels of β-arrestin, which appears to be a crucial modulator for A_1_ receptor availability [90].

In skin inflammation, the effect of A_1_ receptors is less frequently studied, and it mostly focuses on skin diseases related to the neurosystem, such as stress-induced dermatitis. In a study exploring the interplay between stress, A_1_ receptor, and the cutaneous immune response, the role of A_1_ in modulating CHS reactions under stress conditions in mice was investigated [91]. In A_1_ receptor-deficient mice, the CHS response remains unaltered by restraint stress, whereas acute stress enhances CHS responses in wild type mice. It indicates that the presence of A_1_ receptor is essential for stress-related modulation of this immune reaction. The mechanistic link between A_1_ expression within the CNS and the modulation of peripheral immunity provides insights into the complex regulatory networks connecting the CNS and skin and may offer ways to manage stress-induced skin diseases and skin disease-induced CNS dysregulation [91].

### 5.3. A_2A_ Adenosine Receptors

As discussed before, the engagement of adenosine is responsible for a broad spectrum of anti-inflammatory effects in diseases and in animal models [75], which is largely based on A_2A_ receptor activation in immune cells. Its stimulation generally attenuates the inflammatory actions of neutrophils and inhibits cytokine production by eosinophils, monocytes, and T cells, along with inhibition of their activation [92]. This has been proven in mice deficient in A_2A_ receptor expression, as those animals exhibit a heightened inflammatory response, displayed by an altered (hyper-) activation of many kinds of cells [93]. The potential role of this receptor subtype as a pharmacological target in inflammatory diseases, including several different inflammatory skin diseases, has progressively emerged.

In psoriasis, as aforementioned, the A_2A_ receptor subtype is involved in the murine imiquimod-induced model of psoriasis, because imiquimod acts as an A_2A_ receptor antagonist [71,73]. Hence, A_2A_ receptor activation by different agonists is believed to exert therapeutic effects in psoriasis models. For example, the A_2A_ agonist, PDRN, demonstrates therapeutic effects in the imiquimod-induced mouse model of psoriasis by inhibiting the inflammatory response and restoring normal skin architecture. The effect is associated with decreased T cell recruitment and tempered TNF-α, IL-6, and IL-12 expression by LPS-stimulated keratinocytes. Conversely, the use of an A_2A_ antagonist, ZM241385, abolishes these cytokine changes. Importantly, A_2A_ receptor stimulation activates the Wnt signaling pathway and inhibits NF-κB signaling in keratinocytes, thus modulating the secretion of cytokines [83]. Moreover, A_2A_ agonists, such as CV 1808 and forskolin, were found to inhibit chemokine expression in human primary keratinocytes induced by imiquimod [94]. A_2A_ receptors are found to be upregulated in the hyperplastic epidermis, wherein their activation plays a role in modulating pathogenesis by reducing robust inflammation and decreasing the infiltration of leukocytes and the production of cytokines, albeit contributing to keratinocyte proliferation [23]. The upregulation of A_2A_ receptors in psoriatic epidermis becomes the basis of potential A_2A_ receptor stimulation therapy.

In addition to psoriasis, the effect of A_2A_ agonism has also been tested in CHS. In this context, A_2A_/A_2B_ receptor activation has been shown to decrease inflammation in the skin due to reduced T cell infiltration and suppressed DC function(s) and cytokine production. When A_2A_ agonists (CGS 21680) and A_2B_ agonists (BAY60–6583) were applied to the skin before sensitization and challenge, respectively, fewer activated T cells and more anergic cells were induced upon hapten application. This is accompanied by reduced proinflammatory cytokines and chemokines in the respective areas of the skin. These effects were due to reduced numbers of skin migratory DCs in the skin-draining lymph nodes, which were less capable of activating T cells. Interestingly, these effects were long-lasting because the ear swelling reaction of A_2A_ agonist-treated mice was still reduced during rechallenge after 6 weeks [78]. In short, these results support the findings in psoriasis, but provide a more mechanistic explanation.

The topical application of CGS 21680 onto phorbol-induced epidermal hyperplastic skin effectively alleviated symptoms without inducing deleterious atrophic effects generally caused by topical corticosteroids. This is attributed to the induced enhancement of collagen production by fibroblasts through A_2A_ receptor activation [75]. Indeed, activation of adenosine A_2A_ receptors is involved in several events occurring during wound healing, which include inflammation, fibroblast activation, and collagen production. Consistently, treatment with topical selective A_2A_ agonists inhibits the inflammatory response as expected, associated with a large reduction in inflammatory cell infiltrate and a decrease in leukotriene B4 (LTB4) and C-X-C motif chemokine ligand (CXCL)-1 levels and TNF-α, while promoting the growth of dermal fibroblasts [75,95]. Conversely, the use of A_2A_ antagonists have been suggested to prevent irradiation-induced dermal changes, such as fibrosis and atrophy [96].

A_2A_ receptor stimulation augments the synthesis of collagen type I and type III, which are crucial mediators of fibrosis and scarring, via pathways that involve cAMP/PKA/p38 Mitogen-Activated Protein Kinase (MAPK)/Akt signaling. In the case of collagen III, this process also involves β-catenin [97]. Consistently, antagonism of A_2A_ blocks the WNT/β-catenin signaling pathway, thereby reducing dermal fibrosis in diseases such as scleroderma, hypertrophic scarring, and keloid [98]. Given the previously discussed pro-proliferative effect of A_2A_ receptor activation on keratinocytes, future research on A_2A_ agonist therapy must address the dual role of A_2A_ agonists as anti-inflammatory agents but also as promoters of hyperplasia and scar formation. Careful pharmacologic investigations have to balance these opposing effects in possible treatment regimens [24,93].

Direct agonists of A_2A_ receptors are expected to bind specifically A_2A_ receptors and activate defined downstream signaling. However, unexpected effects are frequently caused by their engagement of other adenosine receptors, i.e., by “off-target” effects. In some cases, such “off-target” interactions may either reinforce or attenuate the therapeutic benefits of a respective drug treatment. To reduce side effects caused by “off-target” effects of A_2A_ agonists, a positive allosteric modulator of A_2A_ receptor (AEA061), which enhances receptor affinity and efficacy to endogenous adenosine and inosine, was generated. Its efficacy and safety were tested in the imiquimod-induced psoriasis-like dermatitis mouse model. Both orally and topically given, AEA061 reduced ear swelling, skin thickness, erythema, scale formation, and inflammatory cytokine expression in wild type but not in A_2A_^−/−^ mice. It also successfully ameliorated IL-23-induced psoriasis, as it reduced the secretion of INF-α, IL-23, IL-36α, and IL-17 in γδ T cells [84].

### 5.4. A_2B_ Adenosine Receptors

The affinity of A_2B_ receptors for adenosine is known to be the lowest among all adenosine receptors, suggesting a possible role in pathological conditions where adenosine concentrations rise several-fold. Unlike A_2A_ receptors, the role of A_2B_ receptors in the pathogenesis of inflammatory diseases is less clear and more complicated. In the setting of multiple sclerosis, A_2B_ receptor activation seems detrimental. A_2B_ antagonists CTV-6883 and MRS1754 were reported to alleviate the clinical symptoms of EAE and protect the CNS from immune damage. The effect of CTV-6883 is mediated by inhibiting Th17 differentiation via blocking IL-6 production from dendritic cells. As the A_2B_ receptor-induced activation of IL-6 production is signaled through PLCβ/PKC and p38 MAPK pathways [62], administration of A_2B_ agonists to C57BL/6 mice enhances the development of MAPK-dependent autoimmune uveitis (EAU), with increased Th17 responses. However, these effects were lower in TCR-δ^−/−^ mice, and transfer of γδ T cells into TCR-δ^−/−^ mice partially restored sensitivity to A_2B_ agonists [99]. Hence, the biologic effects induced by stimulation of A_2B_ receptors appear to be dependent on activated Th17 and γδ T cells, which are skewed by specific dendritic cells differentiated by A_2B_ receptor signaling [100]. As γδ T cells as well as different subtypes of DCs reside within the skin and harbor A_2B_ adenosine receptors, these receptors may be a target to effectively modulate skin immunity.

In other inflammatory conditions, it is not fully clear whether A_2B_ receptor activation is pro- or anti-inflammatory; however, more evidence is pointing towards anti-inflammatory functions, as depicted in inflammatory bowel disease and colitis [101,102]. In skin inflammatory diseases less is investigated. As mentioned, topical application of A_2B_ adenosine receptor agonists (BAY60–6583) to the skin reduced ear swelling in CHS reactions by suppressing the activation and migration of skin migratory DCs, thus preventing priming of T cells against haptens [40]. In addition, the anti-inflammatory effects of A_2B_ receptor were found to be less specific. For example, selective A_2B_ agonists, as well as adenosine itself and A_2A_ agonists, are able to inhibit the levels of TNF-α and IL-8 in 12-O-tetradecanoylphorbol-13-acetate (TPA)-stimulated NHEK cells in a concentration-dependent manner; however, this inhibition is not reversed by any of the selective antagonists [23]. Although a reduction in A_2B_ receptor expression was suggested in psoriatic epidermis in vivo, activation of this receptor type by BAY60–6583 shows an antiproliferative effect in NHEK cells in vitro, which is mediated by elevated levels of intracellular calcium. The increased calcium influx arrests the cell cycle in the G1/G0 phase in epidermal keratinocytes, which is a mechanism of some anti-psoriatic drugs such as 1α,25-dihydroxyvitamin D3 or fumaric acid [103]. This may provide a hint that A_2B_ agonism could be a therapy that inhibits both the inflammatory reaction and the over-proliferation of pathologically altered epidermal cells in psoriasis.

In a very recent study [85], based on the antiproliferative and anti-inflammatory effects of A_2B_ receptor activation, the effect of topical application of BAY60–6583 was established in a model of murine epidermal hyperplasia induced by TPA. In detail, topical application of BAY prevented the inflammatory reaction and appearance of skin lesions induced by TPA, minimizing hyperproliferation and acanthosis, as well as the expression of the cell proliferation-specific marker Ki67 in keratinocytes. This is accompanied by reduced leucocyte infiltration markers in skin homogenates, including the chemoattractant CXCL-1 and myeloperoxidase (MPO) activity. Moreover, this effect has been proven to be A_2B_ adenosine receptor-specific since pre-treatment with the respective antagonist (PSB-1115) reversed the anti-inflammatory effects. Cytokines including IL-1β, IL-6, and TNF-α were also reduced by topically applied A_2B_ agonists, although not reversed by PSB-1115. Notably, BAY application preserves epidermal barrier integrity, leading to normalized expression of epidermal barrier proteins including cytokeratin 10 (CK10), CK6, involucrin, filaggrin, and loricrin, which are dysregulated by TPA.

As opposed to reduced expression of A_2B_ adenosine receptors by keratinocytes in lesioned skin, A_2B_ receptor levels are elevated during dermal fibrosis, indicating a pro-fibrotic effect. Therefore, A_2B_ receptor blockade with its antagonist (GS-6201) attenuates dermal fibrosis in mice fibrosis models by reducing levels of arginase-expressing macrophages and myofibroblasts and by decreasing levels of the extracellular matrix proteins fibronectin, collagen, and hyaluronan [28].

Therefore, the activation of A_2B_ receptors constitutes a possible new pharmacological target for the treatment of skin inflammatory diseases characterized by inflammation and epidermal hyperproliferation, such as psoriasis, but it may also lead to fibrosis. These double-faced effects of A_2B_ receptor agonists have to be taken into account when considering them for the treatment of skin conditions.

### 5.5. A_3_ Adenosine Receptors

In psoriasis, A_3_ adenosine receptors represent a new predictive marker as they are known to be overexpressed in the PBMCs of patients with autoimmune inflammatory conditions, e.g., of, RA, and Crohn’s disease. A_3_ receptor expression levels are also higher in the skin from psoriasis patients, whereas healthy cells show low or no receptor expression. This overexpression is directly linked to increased TNF-α levels and is associated with upregulation of NF-κB, which is a key player in the pathogenesis of arthritic diseases [104,105]. The anti-inflammatory effects of A_3_ receptors have therefore been more investigated and utilized in RA patients than in pathological conditions of the skin. Interestingly, the level of A_3_ adenosine receptor expression is inversely related to the disease activity score (DAS), which is used to evaluate disease activity in RA [106]. As far as the function is concerned, in lymphocytes obtained from RA patients, A_3_ receptor activation decreased NF-κB signaling, as well as the production of TNF-α and matrix metalloproteinases.

Considering the anti-inflammatory effect of A_3_ receptor engagement in RA patients by modulating TNF-α and NF-κB, A_3_ agonists for the therapy of RA, as well as for psoriasis, are currently tested in clinical trials or have been evaluated already. Piclidenoson, the oral A_3_ agonist, entered a phase II clinical trial in 2012 and a double-blind phase 3 trial in 2024 in patients with moderate-to-severe plaque psoriasis [107,108]. The treatment improves both Psoriasis Area and Severity Index (PASI) and Physician Global Assessment (PGA) scores. Remarkably, the baseline expression of receptors correlates with the patients’ response to the drug, indicating that A_3_ receptor could potentially serve as a biological marker for predicting the response to CF101 [109]. Moreover, its optimal safety profile renders it a promising candidate for chronic psoriasis therapy. The therapeutic effect was rapid, persistent, and dose-responsive, with higher doses inducing GPCR desensitization in a bell-shaped manner. CF101 exerts its function by inhibiting cytokines such as TNF-α. Also, experiments in HaCaT cells suggest that piclidenoson inhibits the proliferation of keratinocytes by reducing NF-κB signaling and decreasing IL-17 and IL-23 levels (in vitro data) in an A_3_ receptor-dependent mechanism [110]. In addition, CF101 was efficacious in clinical trials on RA [93,109].

Despite the efficacy and safety of CF101 for RA and psoriasis, few cases of CF101 with non-severe adverse effects have been reported, which is likely due to the ubiquitous expression of A_3_ receptors throughout the organism. For instance, a case of hyponatremia was observed in a patient receiving CF101 for advanced hepatocellular carcinoma and moderate hepatic dysfunction [111]; another case was reported in the clinical trial for plaque psoriasis, where infections and gastrointestinal events occurred [112]. Consequently, a therapy with higher selectivity and controllability is required. In a recent study [86], a light-dependent selective A_3_ agonist, MRS7344, was synthesized. This compound, derived from its parent drug MRS5698 and coupled with a photocleavable masking group, a coumarin ether, can dose-dependently inhibit forskolin-mediated cAMP intracellular accumulation, dependent on 420 nm light irradiation. Furthermore, its pharmacological activity was examined in the IL-23 psoriatic mouse model. While MRS7344, used similarly to MRS5698, failed to prevent the IL23-induced ear inflammation, illumination with 420 nm light on one ear after each MRS7344 administration (intraperitoneally) led to a significant reduction in ear inflammation and immune cell infiltration. However, no comparable anti-inflammatory effect was observed on the contralateral, unilluminated ear of the animals treated with MRS7344. This suggests a lack of systemic diffusion of MRS5698 upon photorelease, thereby reducing the adverse effects of MRS7344 and allowing for tissue- and time-controlled administration in a light-dependent fashion. The development of A_3_ receptor light-dependent drugs offers a promising therapeutic approach for inflammatory skin diseases by utilizing light to activate drugs, akin to photodynamic therapy [113], achieving high efficacy while avoiding unwanted immunosuppressive side-effects in other organs.

## 6. Overview of Current Treatments Interacting with Adenosine Pathways

Non-steroidal anti-inflammatory drugs (NSAID) that are applied to treat psoriatic arthritis and to relieve pain in skin diseases are thought to involve adenosine pathways. For instance, high doses of acrtylsalicylic acid (ASA) in rheumatoid arthritis patients were shown to elevate adenosine signaling [114]. Another NSAID, sulindac, generates an active metabolite, sulindac sulfide, which competitively inhibits ENT1, an adenosine transporter, thus prolonging the activity of intercellular adenosine by inhibiting its uptake [115].

Glucocorticoids are the first-line therapy for most inflammatory skin diseases. Although adenosine-dependent mechanisms for the action of glucocorticoids on leukocytes were not identified [116], adenosine receptors may be involved as a secondary mechanism of glucocorticoids in treating inflammation. For instance, activation of the A_3_ adenosine receptors by glucocorticoids promotes the survival of anti-inflammatory human monocytes [117].

Topical application of rapamycin in an imiquimod-induced psoriasis model not only alleviates inflammatory responses in psoriatic skin lesions by inhibiting the mTOR pathway, but also normalizes the expression of epidermal differentiation-related markers [118]. One of the possible mechanisms was found to block adenosine uptake by binding to the adenosine transporter molecule ENT1, which consequently increases intercellular concentrations of adenosine and signaling thereof [119].

Conventional systemic medications like oral methotrexate (MTX) represent first-line and cost-effective options for treating psoriasis. However, the long-term use of MTX in certain high-risk patients is limited by various systemic side effects, including liver toxicity and bone marrow suppression [120]. The classical mechanism underlying MTX’s anti-inflammatory actions involves its functions as an antiproliferative agent, inhibiting dihydrofolate reductase and consequently blocking DNA synthesis and cell mitosis in rapidly dividing cells [121]. Intriguingly, it has been revealed that many of MTX’s effects are mediated by adenosine accumulation following MTX administration [75]. MTX induces the release of adenosine in vitro and in vivo in animal models of inflammation as well as in patients with rheumatoid arthritis [122]. This process is facilitated by the inhibition of 5-aminoimidazole-4-carboxamide ribonucleotide (AICAR) transformylase, resulting in elevated intracellular AICAR levels, subsequent inhibition of AMP and adenosine deaminase, and ultimately, accumulation in extracellular adenosine [123,124]. The relevant studies indicate that antagonists of A_2A_ and A_3_ adenosine receptors can reverse or prevent the anti-inflammatory effects of MTX [125].

Adenosine is also highlighted as a key active ingredient of Isatis tinctoria L (PLG), a medical herb from the roots of Isatis indigotica Fort. Oral administration of PLG is reported to attenuate skin lesions in 2,4-Dinitrochlorobenzene (DNCB)-induced CHS models, with reduced cytokine production via NF-κB signaling in skin lesions and activated keratinocytes. Adenosine, as well as another compound, i.e., epigoitrin in PLG, have a direct inhibitory effect on chemokine production in TNF-α/IFN-γ-induced keratinocytes [126]. It offers the possibility that adenosine has broad and uninvestigated therapeutic effects, even in herbal medicine.

Biologic treatments are designed to target the altered inflammatory milieu and have been shown to be effective in moderate to severe psoriasis, when used after unsuccessful attempts with other systemic treatments [24]. The widely used anti-platelet and vasodilator drug, dipyridamole 21, which was initially shown to inhibit cyclic nucleotide phosphodiesterases (PDEs), probably has its main effects through ENT1 inhibition, leading to activation of adenosine receptors by increasing local extracellular adenosine concentrations [127,128]. Interestingly, the direct inhibitor of PDE4 (Apremilast) blocks the degradation of cAMP and leads to increased cAMP, similar to the effect triggered by A_2A_/A_2B_ activation. Therefore, the two kinds of specific drugs, targeting different pathways upstream of cAMP, may exert synergistic effects on the therapy of some inflammatory conditions, and indeed, A_2A_ agonism is reported to strengthen the effect of apremilast by inhibiting TNF-α [129].

## 7. Conclusions

Influencing adenosine signaling emerges as a compelling strategy for mitigating undesirable immune responses due to the widespread expression of the adenosine receptors and their ability to regulate the function of immune and non-immune cells. Firstly, it should be of interest to determine whether a tissue- and/or disease-specific adenosine receptor expression pattern could potentially serve as a biomarker in the trajectory of skin pathology. As a consequence, strategies to enhance the inhibitory actions of adenosine receptors could exhibit strong anti-inflammatory effects, while blocking those receptors may stimulate immunity during cancer treatment.

Nonetheless, it is crucial to understand whether and how the signaling pathways triggered by adenosine receptor agonists and antagonists are indeed adenosine receptor-specific, as all four adenosine receptors have partially opposed effects. These Janus-faced actions of adenosine receptors may limit the efficacy of small inhibitory molecules, as different compositions of inhibitory and stimulatory adenosine receptors may outcompete each other for the desired actions. Moreover, the ubiquitous expression of adenosine receptors by many different cell types and tissues makes it complicated to use systemic adenosine receptor modulating agents to specifically target one organ or disease, respectively.

Nevertheless, once the mechanistic actions of adenosine are understood, the skin, as opposed to internal organs, offers the possibility for topical treatment. Therefore, novel therapies for hyperproliferative diseases may emerge soon. One example, based on the dual roles of A_2A_ and A_2B_ adenosine receptors in modulating keratinocyte proliferation, may be psoriasis. Future investigations should elucidate the feasibility of utilizing A_2A_ agonists as potential agents for reducing inflammation and(or) hyperplasia in these conditions.

## Figures and Tables

**Table 1:** Ongoing animal studies of adenosine receptor ligands.

Ligands	Receptor Selectivity	Mouse Model	Effect	Reference
KN-62	P2X_7_ antagonist	CHS	Reduced reaction and IL-1 secretion by DCs	[80]
A438079	P2X_7_ antagonist	croton oil-induced oedema	Impair of croton oil-induced oedema; reduced IL-1β production and neutrophil infiltration	[82]
A438079	P2X_7_ antagonist	ATP analog and E-NTPDase inhibitors-induced psoriasiform dermatitis	Block of psoriasiform dermatitis and inflammatory response	[76]
PDRN	A_2A_ agonist	imiquimod-induced mouse model	Inhibition of inflammatory response and restoration of normal skin architecture, decreased T cell recruitment, and a shift towards an anti-inflammatory cytokine profile	[83]
CGS 21680	A_2A_ agonist	CHS	Less-activated T cells and more anergic cells; reduced proinflammatory cytokines and chemokines in inflamed ear; reduced functional skin migratory DCs, which are also less functional	[78]
CGS 21680	A_2A_ agonist	phorbol-induced epidermal hyperplasia	Reduction in epidermal hyperplasia and promotion of collagen synthesis normalization of epidermal structure and enhancement of fibroblast proliferation in the dermis reduction of chemotactic mediator expression and NF-κB inhibition	[75]
AEA061	positive allosteric modulator of A_2A_	imiquimod-induced psoriasis-like dermatitis mice model	Reduced ear swelling, skin thickness, erythema, scale formation, and inflammatory cytokine expression	[84]
BAY60–6583	A_2B_ agonist	CHS	Reduced ear swelling; suppressed activation and migration of skin migratory DCs	[78]
BAY60–6583	A_2B_ agonist	TPA-induced epidermal hyperplasia	Reduced skin inflammation; reduced leucocytes infiltration; preserved epidermal barrier integrity	[85]
MRS5698	Photosensitive A_3_ agonist	IL-23 mouse model of psoriasis	Reduced skin swelling; cAMP reduction	[86]

CHS: contact hypersensitivity; DCs: dendritic cells; IL: interleukin; cAMP: cyclic adenosine monophosphate; TPA: 12-O-Tetradecanoylphorbol-13-acetate.

**Table 2:** Ongoing clinical trials of adenosine receptor ligands.

Ligands	Receptor Selectivity	Indication	Phase	Reference
Poclidenoson	A_3_ agonist	Psoriasis	3	NCT00428974
Poclidenoson	A_3_ agonist	Rheumatoid arthritis	3	NCT00428974
